# Computation of simple invariant solutions in fluid turbulence with the aid of deep learning

**DOI:** 10.1007/s11071-025-11773-1

**Published:** 2025-09-18

**Authors:** Jacob Page

**Affiliations:** School of Mathematics & Maxwell Institute for Mathematical Sciences, University of Edinburgh, EH9 3FD, Edinburgh, UK

**Keywords:** Fluid dynamics, Turbulence, Machine learning

## Abstract

The dynamical systems view of a turbulent fluid flow provides a tantalizing connection between the self-sustaining nonlinear mechanics of turbulence and its more well-known statistical properties, and promises to open up new avenues in our ability to understand, predict and control complex fluid motion. However, successful application of these ideas to a high Reynolds number (*Re*) problem requires the discovery and convergence of an expansive library of simple invariant solutions (e.g. equilibria, periodic orbits). The key challenge for the field has been that algorithms to compute dynamically relevant structures struggle for a variety of reasons outside of the weakly turbulent regime. It is here that ideas from deep learning have started to show promise, and this review describes how various techniques from the machine learning community have accelerated progress. First, the use of autoencoders – neural networks which perform a nonlinear analogue to PCA – will be described. There is compelling evidence that the low-order representations of the flow learned by these models are closely connected to the unstable simple invariant solutions embedded in the turbulent attractor. As such, these representations can be used to measure shadowing of periodic solutions, to parameterize reduced order models and to estimate manifold dimension. The other key technique adapted from deep learning reviewed here is the advance in high-dimensional, gradient-based optimization that has been driven by the requirements of neural network training. To exploit these tools, the search for simple invariant solutions is converted to a hunt for minima of a scalar loss function, and gradient computation is performed efficiently within a fully differentiable flow solver. Using forced, two-dimensional turbulence as a test case, these new methods reveal an order of magnitude more solutions than has been possible using earlier approaches and converge periodic orbits where previous methods have been ineffective. An assessment will be made as to what the large set of new exact solutions says about the ‘dynamical systems’ exercise in general and the prospects for application at high *Re*.

## Introduction

Viewing turbulent fluid motion as an orbit in a very high-dimensional state space was an idea first described over 75 years ago by Hopf [35]. In this framework a turbulent trajectory for a statistically steady configuration is thought of as a ‘pinball’ bouncing between *simple invariant solutions* [16, 67], or *exact coherent structures* (ECS), terminology which encompasses (relative) equilibria, (relative) periodic orbits and tori. A chaotic turbulent orbit transits between these objects, shaped by their stable and unstable manifolds [20, 29, 43].

This viewpoint of turbulence is a compelling one for several reasons:

- Each exact coherent state encapsulates a self-sustaining process relevant to the turbulent dynamics, and can be used to gain a mechanistic understanding of the flow processes [15, 30, 42, 61].
- The ‘pinball’ view of turbulence is an alternative approach to low-order modeling without loss of spatial complexity; the ‘reduced order model’ being a Markov chain with the states being ECS with a full range of scales [73, 84].
- There is scope to use a “complete” set of ECS to relate the statistics of the turbulence to the statistics of periodic orbits via application of ‘periodic orbit theory’, which states that a statistic of a chaotic attractor in a uniformly hyperbolic dynamical system can be expressed as a weighted sum of the same statistic evaluated for each periodic solution [3, 4, 19]. The weights depend on local stability properties of the periodic orbits.

The notion of ‘completeness’ of an ECS library – which is a terminology reserved for periodic orbits – requires the existence of a symbolic dynamics, in which state space is partitioned into disjoint regions and trajectories written as a sequence of symbols which describe sequentially the regions visited. Periodic orbits then become periodic symbol sequences, and a library of these solutions is complete up to length $N_{sym}$ if all periodic orbits with sequence length $< N_{sym}$ have been found [19]. The final point in the list above would in principle allow one to connect statistical properties of e.g. the inertial range cascade to self-sustaining dynamical processes.

With this motivation, a great deal of effort has been made to find ECS, with an understandable focus on periodic orbits, in a wide variety of flow geometries and with various additional physical effects [15, 20, 27–29, 41–43, 52, 61, 62, 72, 83]. Historically, the search for periodic orbits has relied on ‘recurrent flow analysis’ – a search for near-closed loops on a turbulent orbit (typically measured using an $L_2$-norm on the vorticity field) [15, 83]. This has proven to be quite effective at low Reynolds numbers (*Re*), but near recurrences become increasingly unlikely at high-*Re* as the underlying periodic orbits become more unstable. Moreover, measuring distance between vorticity fields (say) with an $L_2$ norm is unlikely to be best measure of similarity of states. As a result, alternative search approaches have been proposed, including the use of different observables in a recurrent flow analysis [71, 77] or the use of dynamic mode decomposition to identify the fundamental frequency of a nearby periodic solution in the state space [65, 72] without requiring a near-closed loop in the observations.

There are natural opportunities for the adoption of ideas from the machine learning community within the search for ECS, and this paper reviews some recent work on two particular ideas. One has to do with using deep neural networks to learn efficient reduced order representations of turbulence [57, 66, 70, 71, 86]. That these models can be effective at data compression of turbulence is not surprising given the success of convolutional neural networks in various tasks related to images [31, 54, 55]. While autoencoders are helpful in a variety of contexts, e.g. identifying appropriate coordinates for reduced-order models [14], the focus here is on their utility to measure similarity between flow snapshots – something which is useful both in better flagging near recurrences but also in identifying when a turbulent orbit is near a known ECS.

The other idea from the machine learning community is the combination of effective gradient-based optimization with *fully-differentiable* dynamical solvers. The “fully-differentiable” terminology here implies the ability to backpropagate gradients through trajectories (repeated application of a time-stepper) of the system under consideration *without* the use of adjoints. This is accomplished using automatic differentiation [6]. In fluid dynamics this approach has allowed for the training of more effective neural-network-based turbulence models, improving stability by requiring that the predictions can be unrolled in time [59, 82]. On a much grander scale, similar ideas have been highly effective in training models to parameterize e.g. cloud formation in general circulation models [51]. However, the focus in this paper is not on the training of models, but more on the utility of gradient-based optimization as a strategy to find exact solutions of the Navier-Stokes equations [73], which will prove to be a highly effective method to avoid the need for near recurrences on turbulent orbits. The approach relies on differentiability of the underlying solver [21, 50], the flexibility to design bespoke solution-targeting loss functions and the speed and efficiency of optimization on a GPU. These features have also been exploited in other contexts such as mixing of non-Newtonian fluids [1] and controlling non-equilibrium systems [23].

While the use of machine learning in other problems relevant to fluid dynamics is also touched on briefly here, this review is focused specifically on the utility of these new computational methods in the search for unstable simple invariant solutions. The new methods lead to the convergence of large numbers of new ECS that were completely missed by previous methods, and the assembly of an expansive library of ECS raises some interesting questions on the utility of periodic orbits in reproducing statistics at high *Re*, whether via application of periodic orbit theory or through a data-driven methodology.

The remainder of this paper is structured as follows. In §2 the governing equations are introduced, along with the flow configuration used to illustrate many of the new techniques. In §3 the use of autoencoders in the search for ECS is explored, which relies on ‘latent Fourier analysis’, an interpretability technique for models trained in systems with continuous symmetries. In §4 the use of gradient-based optimization around a fully differentiable solver is reviewed as a technique to search for ECS with specific properties, before the utility of the solutions for predicting statistics is discussed in §5. Finally, conclusions are provided in §6.

## Simple invariant solutions in fluid dynamics

### Governing equations

Most of the examples described in the following sections are governed by the incompressible Navier-Stokes equations,

1a $$\begin{aligned} \varvec{\nabla } \cdot {\textbf{u}}&= 0, \end{aligned}$$

1b $$\begin{aligned} \partial _t {\textbf{u}} + ({\textbf{u}} \cdot \varvec{\nabla })\textbf{u}&= -\varvec{\nabla } p + \frac{1}{Re} \varDelta {\textbf{u}} + {\textbf{f}}, \end{aligned}$$

where ${\textbf{u}} = (u,v)$ is a two-dimensional velocity field (though nothing about the approach in general is restricted to 2D flows) and *p* is the pressure. In the examples below a body force ${\textbf{f}}$ drives the flow. The Reynolds number $Re := U L /\nu$, where *U* and *L* are reference velocity and length scales, while $\nu$ is the kinematic viscosity of the fluid.

Many flows of interest have continuous spatial symmetries – for instance three-dimensional computations of periodic channels are equivariant under arbitrary two-dimensional horizontal shifts. The two-dimensional flow configuration here is equivariant under shifts in the horizontal (*x*) direction. As a result, the simple invariant solutions of (1) sought are typically *relative* periodic orbits (RPOs), for which

2 $$\begin{aligned} {\textbf{F}}({\textbf{u}}, T, \alpha ) = {\mathscr {T}}^{\alpha } \varvec{\varphi }_T ({\textbf{u}}) - {\textbf{u}} = {\textbf{0}}, \end{aligned}$$

where ${\mathscr {T}}^{\alpha } : {\textbf{u}}(x,y,t) \rightarrow \textbf{u}(x+\alpha ,y,t)$ performs translation by an amount $\alpha \in {\mathbb {R}}$ and $\varvec{\varphi }_t$ is the time-forward map (we avoid the term ‘flow’ due to its association with fluid motion) associated with (1). Equation (2) simply means that the velocity field ${\textbf{u}}$ returns to the same point in state space after time advancement by a period *T*, modulo a shift in the *x*-direction. Relative equilibria, also called traveling waves, are solutions of (2) with arbitrary *T* where the shift $\alpha$ is then related to the wavespeed via $c = \alpha / T$. In a system without continuous symmetries, we would expect to find exact periodic orbits and equilibria without shifts.

Typically, solutions $({\textbf{u}}, T, \alpha )$ of (2) are found via a high-dimensional Newton-Raphson algorithm, with update directions determined by GMRES [81] to avoid explicit computation and inversion of the Jacobian. In most cases, a hookstep constraint is required to control the size of the update – something which is essential in the early stages of convergence [15, 83]. There has been a great deal of attention given to the problem of selecting ‘good’ initial guesses $({\textbf{u}}_0, T_0, \alpha _0)$ to seed the Newton algorithm with [72, 77].

### Kolmogorov flow

Monochromatically forced, two-dimensional flow on the two-torus (‘Kolmogorov flow’) will be used as an illustrative example throughout this paper. This flow has been a testing ground for various simple invariant solution search techniques over the past decade or so [15, 62, 70, 71, 73, 74]. The early study by Chandler & Kerswell [15], in which *O*(50) periodic orbits were found in a recurrent flow analysis over hundreds of thousands of advective time units, will be used frequently as a point of comparison.

The Kolmogorov flow considered here is driven by a force $\textbf{f}^* = \chi ^*\sin (2\pi n\, y^* /L^*) \hat{{\textbf{x}}}$ (here the asterisk indicates dimensional variables, with $L^*$ the dimensional domain height and $\chi ^*$ the amplitude of the force). The number of forcing waves is set at $n=4$, and the forcing amplitude is used to define a reference velocity scale, $\sqrt{\chi ^* L^*/2\pi }$, while the box sets the reference lengthscale, $L^*/2\pi$. As a result, the Reynolds number is $Re := \sqrt{\chi ^* (L^*/2\pi )^3} / \nu$. Equal aspect-ratio domains are considered throughout, hence the dimensionless box size is $L_x = L_y = 2\pi$.

This particular Kolmogorov flow is equivariant under continuous translations in *x*, as well as discrete vertical shift-reflects and rotations [15, 24]. Discrete symmetries are not considered/searched over when looking for relative periodic orbits. This omission should be corrected in future work: it is straightforward to add shift-reflects and/or rotations to equation (2) (and the optimization approaches introduced later). These discrete symmetry operations are ‘hard coded’ and are not determined as part of the solution, unlike the horizontal shift, $\alpha$. The continuous symmetry is central to the neural network interpretability algorithm described below.

It is convenient to take the curl of (1b) and work with the out-of-plane vorticity, $\omega := \partial _x v - \partial _y u$,

3 $$\begin{aligned} \partial _t \omega + {\textbf{u}} \cdot \varvec{\nabla }\omega = \frac{1}{Re}\varDelta \omega - n\cos ny . \end{aligned}$$

The velocity field can be obtained from the vorticity via solution of $\varDelta \psi = -\omega$, where $u = \partial _y \psi$, $v=-\partial _x\psi$. One feature of Kolmogorov flow is that an arbitrary constant background velocity, $(U, V)=\text {constant}$, can be added to the problem. The addition of *U* is of no consequence, and the problem is equivalent to the case $U=0$ when viewed in the appropriate Galilean reference frame. However, the addition of a background *V* results in the cross-stream advection of vorticity and does fundamentally change the problem. The addition and removal of a background *V* occurs naturally in the optimization for periodic orbits described in §4; many solutions found for finite *V* can be continued via homotopy back to the case of interest, $V=0$.

## Autoencoders

We first consider the idea of learning low-dimensional representations of the inertial manifold of solutions to the governing equations.

### Reduced-order representations of turbulence

The computational challenge of simulating turbulence drives the need for robust low-order models to enable rapid prediction and for use in model-based control [78]. Historically, a good deal of attention has been given to a representation of the flow in terms of leading POD ($\equiv$PCA) modes [34]. The advantages of this approach are that (i) computation of the basis is straightforward given set of flow snapshots, (ii) the modes are orthogonal and ordered by their ‘energy’ and, most usefully, (iii) the basis is *interpretable* – individual modes look like velocity fields and can be visualized individually.

On the other hand, nonlinear models – specifically neural networks in ‘autoencoder’ configurations – can be much more efficient at data compression, though this benefit is gained at the expense of interpretability of the low-order representation; individual neurons within a neural network architecture do not in general correspond to a single, well-defined feature or structure in the flow [68].

Autoencoders are approximations to the identity function. For the Kolmogorov flow considered here, we seek an autoencoder such that ${\mathcal {A}}_{\varvec{\varTheta }}(\omega ) \approx \omega$. If the flow is discretized on *N* total gridpoints, then $\mathcal A_{\varvec{\varTheta }}: {\mathbb {R}}^N \rightarrow {\mathbb {R}}^N$. In the examples here, the autoencoder’s structure consists first of an ‘encoder’, ${\mathcal {E}} : {\mathbb {R}}^N \rightarrow {\mathbb {R}}^m$, with dimensionality reduction $m \ll N$, before a ‘decoder’, ${\mathcal {D}}: {\mathbb {R}}^m \rightarrow {\mathbb {R}}^N$, returns the approximation to the original field, i.e. ${\mathcal {A}}_{\varvec{\varTheta }} \equiv {\mathcal {D}} \circ {\mathcal {E}}$. To construct an autoencoder using a deep neural network requires specification of a large number of parameters – the weights of the network, $\varvec{\varTheta }$. In the examples discussed here the number of parameters is typically on the order of a million. These parameters are determined via gradient based optimisation of a loss of the form,

4 $$\begin{aligned} {\mathscr {L}}_{AE}(\varvec{\varTheta }) = \frac{1}{N_S}\sum _{j=1}^{N_S}\Vert \omega _j - \mathcal A_{\varvec{\varTheta }}(\omega _j)\Vert ^2, \end{aligned}$$

where $N_S$ is the number of snapshots in the ‘training’ dataset. Other terms can be added for specific purposes, and some examples of this are discussed below. For brevity the subscript $\varvec{\varTheta }$ will be suppressed in the following text.

The use of autoencoders for dimensionality reduction of turbulent flow was first described in [66], where the connection to the linear PCA was also established (neural networks with linear activations which minimize (4) are equivalent to a projection onto *m* PCA modes). One particularly exciting approach associated with dimensionality reduction via autoencoders is the ability to discover coordinate systems which enable the discovery of sparse dynamical models of complex phenomena – small systems of differential equations from which the original high-dimensional state can be recovered by the decoder [14]. This is accomplished via additional terms in the loss connected to model discovery (the SINDy algorithm, see [11]). Other work has imposed more stringent restrictions – e.g. that the latent ($\equiv$ ‘embedded’) dynamics are linear [63].

In the context of the dynamical systems approach to turbulence, autoencoders have been used to probe the dimension of the inertial manifold in spatiotemporally chaotic systems. For example, Linot & Graham [57] were able to identify the dimension of the inertial manifold for the Kuramoto-Sivashinky (KS) equation in the weakly chaotic regime via incremental reduction in the size of the embedding vector. The need to update and re-train the architecture incrementally was removed in [86], which adapted the empirical observations of [40] – that a series of learned linear operations applied to the embedding naturally lead to a low-rank representation – to a turbulent Kolmogorov flow. Furthermore, autoencoders have been combined with other time-series models to perform time-advancement in the latent space [76], while similar ideas have been applied [58] in a weakly turbulent Couette flow to build a low-order ‘neural differential equation’ which approximates the time evolution. The neural differential equation was then used to find periodic orbits of the original system of equations. In some recent work, embeddings of KS periodic orbits have been combined to find new orbits which shadow multiple solutions in sequence [7].

Many of these examples exploit symmetry reduction (e.g. Fourier mode slicing for continuous symmetries [12]) to avoid the need to design for or learn the symmetry group. Symmetry reduction is not performed in the approach described below, ‘latent Fourier analysis’, which was introduced and applied in a series of papers [70, 71, 73] and which relies on a learned embedding of the symmetry to extract a physically interpretable set of basis vectors for embeddings. Interpretability is an active area of research in machine learning in general [68, 80], and there are other exciting ideas being developed in neural networks for the physical sciences (e.g. see [44, 64]) which are beyond the scope of the current paper.

The specific autoencoders used in the discussion below were introduced in [71], and have encoder and decoder modules which are deep convolutional neural networks [55]. These architectures are the natural choice here due to the fact that they respect the translation equivariance of local features in the vorticity field. Some design choices were made in the models introduced in [71], for instance use of ‘dense’ layers in which the outputs of sequential convolutions are concatenated to build a richer set of feature maps [31, 36]. Similar to developments in machine learning in general, these choices are based on empirical observation of improvement rather than rigorous results, but generally all the models described have relative reconstruction errors, $\varepsilon := \Vert \omega - \mathcal A(\omega )\Vert _2 / \Vert \omega \Vert _2$ on the order of a few percent. The neural network architecture is summarized in Appendix A.

### Latent Fourier analysis

A strategy was proposed in [70] to identify physically significant directions in the inner-most representation (the ‘embedding’) within an autoencoder. The approach, which was dubbed ‘latent Fourier analysis’, exploits symmetries in the physical problem to find an interpretable set of basis vectors in the latent space of the autoencoder. These basis vectors can be individually decoded into vorticity fields, and identify common ‘recurrent patterns’ in the flow.

The Kolmogorov flow discussed extensively here is equivariant under translations in the streamwise direction, *x*:

5 $$\begin{aligned} {\mathscr {T}}^s : \omega (x,y,t) \rightarrow \omega (x+s,y,t). \end{aligned}$$

To interpret our embeddings, $\{{\mathcal {E}}(\omega _i)\}$, we seek to construct an operator that performs shifts *in the latent space*,

6 $$\begin{aligned} {\textbf{T}}_{\alpha } {\mathcal {E}}(\omega ) := {\mathcal {E}}(\mathscr {T}^{\alpha } \omega ). \end{aligned}$$

Numerically, an approximate shift operator is determined by a least-squares fit over the embedding dataset:

7 $$\begin{aligned} \widehat{{\textbf{T}}}_{\alpha } = \text {arg min}_{\textbf{T}_{\alpha }}\sum _{j=1}^{N_S} \Vert {\textbf{T}}_{\alpha } \mathcal E(\omega _j) - {\mathcal {E}}({\mathscr {T}}^{\alpha } \omega _j)\Vert _2^2, \end{aligned}$$

which has solution $\widehat{{\textbf{T}}}_{\alpha } = {\textbf{E}}^+ {\textbf{E}}'$, where the ‘$+$’ superscript indicates a Moore-Penrose pseudo inverse, while ${\textbf{E}}$ has columns ${\mathcal {E}}(\omega _j)$ and ${\textbf{E}}'$ has columns ${\mathcal {E}}(\mathscr {T}^{\alpha }\omega )$. Here $N_S$ now denotes the number of snapshots in a separate ‘test’ dataset which was not shown to the autoencoder when it was trained. Algorithmically this approach is equivalent to dynamic mode decomposition which seeks a best-fit linear operator to shift observables forward in time [79].

If the shift is selected such that $\alpha = 2\pi / n$, where $n\in {\mathbb {N}}$, then *n* applications of the shift operator return a snapshot to its original orientation, ${\textbf{T}}_{\alpha }^n \mathcal E(\omega ) = {\mathcal {E}}(\omega )$. The eigenvalues of $\widehat{{\textbf{T}}}_{\alpha }$ are therefore expected to approximate the $n^{\text {th}}$ roots of unity.

There is no reason a-priori to suspect that translational equivariance is embedded in a linear manner for any given network architecture. However, the purely convolutional architectures of [71] do retain discrete translational equivariance up to the resolution of the coarsest feature map: Since dimensionality reduction is done between convolutions by a pooling operation, each ‘row’ of the inner-most feature maps corresponds to a subdomain of the original image. For example, if the feature maps which make up ${\mathcal {E}}$ are of width $M \in {\mathbb {N}}$, then each row *j* of the embedding corresponds to physical features found for $x \in [2\pi j / M, 2\pi (j+1)/M]$ and translations in physical space by $2\pi / M$ correspond exactly to a permutation of the rows in ${\mathcal {E}}$. Shifts by a smaller amount $\alpha < 2\pi /M$ are therefore accomplished approximately in the embedding, and the success of the approach is an empirical observation. However, we have observed that even architectures without inherent translational equivariance do appear to settle on a linear embedding of translational symmetry if trained for long enough and on a rich enough dataset [70, 71].

**Fig. 1:**
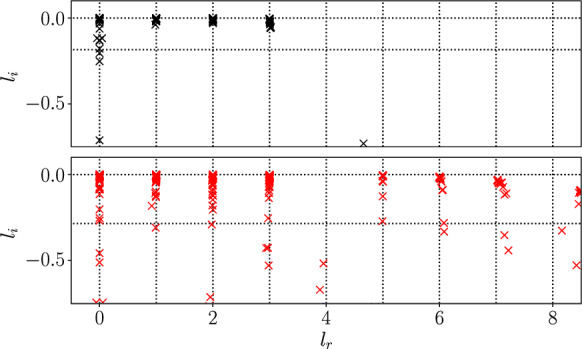
Eigenvalues of the latent shift operator, $\widehat{{\textbf{T}}}_{\alpha }$ at $Re=40$ (top, shift $\alpha = 2\pi /n$, $n=11$ for a neural network with a 128-dimensional embedding) and $Re=100$ (bottom, shift $\alpha = 2\pi /n$, $n=17$ for a neural network with a 512-dimensional embedding). The eigenvalues are visualized in terms of their latent wavenumber $l = -n \log \varLambda / 2\pi$, where we expect $\text {Im}(l) = 0$ in a perfect shift operator. Vertical dashed lines indicate $l_r \in {\mathbb {Z}}$ while the horitzonal dashed line is the threshold corresponding to $\varLambda = 0.9$

Some example eigenvalue spectra for two latent shift operators ${\textbf{T}}_{\alpha }$ are reported in figure 1 for autoencoders trained in Kolmogorov flow at $Re=40$ and $Re=100$. The eigenvalues are visualized as latent wavenumbers *l*, which are obtained from $\{\varLambda _j\}$ by writing $\varLambda = \exp (2\pi i l /n)$. One expects $l \in {\mathbb {Z}}$, and this is indeed approximately observed in the spectra in figure 1. Note that eigenvalues are found only for some fixed range of $|l| \le l_{\text {max}}$: in practice the shift $\alpha$ is incrementally reduced until no new *l* are observed beyond $l_{\text {max}}$ in the spectrum. In the examples reported in figure 1, only three non-zero latent wavenumbers are required at $Re = 40$, while $l_{\text {max}} = 7$ at $Re=100$.

The small number of latent wavenumbers found is possible as the network learns a data compression based on a series of fundamental patterns of horizontal periodicity $2\pi / l$, which tie together physical wavenumbers $k = ql$, where $q\in {\mathbb {Z}}$. This effect results in degeneracy in the eigenspaces which is clearly observed in the clusters of eigenvalues around integer *l* in figure 1.

We now consider only the directions which best retain streamwise-translation invariance, ignoring modes for which $|\varLambda | <0.9$ (say). Consider the embedding of a snapshot subject to arbitrary shift *s*, written as an expansion in the remaining eigenvectors of $\widehat{{\textbf{T}}}_{\alpha }$:

8 $$\begin{aligned} {\mathcal {E}}({\mathscr {T}}^s \omega ) \approx \sum _{l=-l_{\text {max}}}^{l_{\text {max}}} \left( \sum _{k=1}^{d(l)} {\mathcal {P}}_k^l({\mathcal {E}}(\omega )) \right) e^{i l s}. \end{aligned}$$

In this expression, the inner sum is over the degenerate eigenspace associated with each *l* (*d*(*l*) is the geometric multiplicity) and the projection ${\mathcal {P}}_k^l$ is defined as

9 $$\begin{aligned} {\mathcal {P}}_k^l({\mathcal {E}}(\omega )) := \left[ \varvec{\xi }_k^{l\dagger *} \cdot {\mathcal {E}}(\omega ) \right] \varvec{\xi }_k^l, \end{aligned}$$

where $\varvec{\xi }_k^l$ is one of *d*(*l*) eigenvectors for which $\widehat{{\textbf{T}}}_{\alpha }\varvec{\xi }_k^l = \varvec{\xi }_k^l\exp (2\pi i l /n)$ and $\varvec{\xi }_k^{l\dagger }$ the corresponding biorthonormal adjoint mode.

**Fig. 2:**
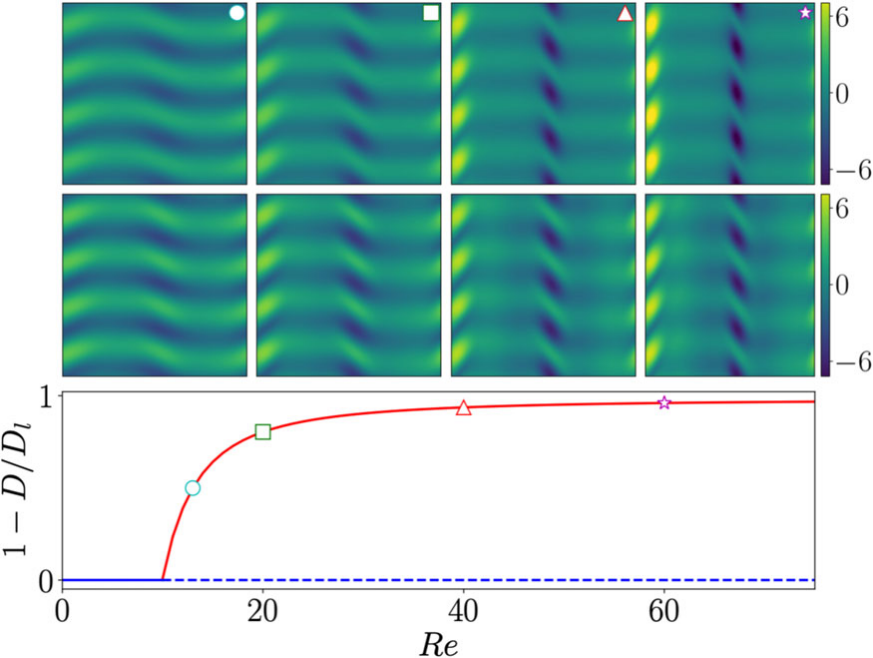
Comparison of the decode of leading latent modes and a known equilibrium solution in Kolmogorov flow, for an autoencoder with an $m=64$ dimensional latent space (architecture introduced in [71] and described in the Appendix). Shown are out-of-plane vorticity contours for the known equilibrium (top) and the decode of the leading $l=0$, $l=1$ combination described in the text (middle – mode amplitudes are determined via encoding the equilibrium itself and setting all other latent modes to zero). The comparison is performed at the points identified with symbols on the solution curve (bottom) visualized by the departure of the dissipation rate from the laminar value, $D_l$. A similar figure was generated in [70] for a completely different neural network, indicating the robustness of this effect

Equation (8) is a ‘latent Fourier transform’ of the embedding ${\mathcal {E}}(\omega )$. Individual/combinations of latent Fourier modes in particular eigenspaces *l* can be decoded and visualized, i.e. one can compute

10 $$\begin{aligned} \omega _{l} = {\mathcal {D}}\bigg (\underbrace{\sum _{k\in S_0}\mathcal P_k^0({\mathcal {E}}(\omega ))}_{l=0\text { contribution}} + \left[ \sum _{k\in S_l}{\mathcal {P}}_k^l({\mathcal {E}}(\omega )) + \text {c.c.} \right] \bigg )\nonumber \\ \end{aligned}$$

where $S_l$ contains a subset of modes from eigenspace *l* – note that the $l=0$ space must always be included for physically realistic outputs [70].

In references [70, 71] an SVD is performed in each eigenspace, and individual SVD modes are then decoded to reveal a corresponding pattern in physical space. One example for an autoencoder with an $m=64$-dimensional embedding trained at $Re=40$ [71] is shown in figure 2. This figure shows the bifurcations of the first non-trivial equilibrium from the laminar state, viewed in terms of its dissipation *D* ($D_l$ is the laminar value); above which the non-trivial equilibrium is shown at various points on the bifurcation curve (see filled symbols). Included below the equilibria are decodes of a superposition of the leading SVD mode from each of the $l=0$ and $l=1$ subspaces (10) – i.e. the physical pattern is constructed from just *two* latent directions. The pair of modes are able to qualitatively reproduce the non-trivial equilibrium across the solution curve, despite that fact that (i) no turbulent snapshot in the dataset contains this structure and (ii) training was conducted at fixed $Re=40$. A similar analysis was performed for an entirely different network architecture in reference [70], indicating the robustness of this embedding ‘strategy’ learned by the networks.

The association of this particular structure – which is of dynamical significance in the flow as many periodic orbits can be traced back to this solution through a sequence of bifurcations [15] – with a particular direction in the latent space, and the ability to reproduce its nonlinear deformation over the bifurcation curve via a linear superposition of just two embedding vectors is in sharp contrast to linear model reduction strategies (e.g. PCA modes here are just Fourier modes).

The dynamical connection is apparently not restricted to just the leading $l=1$ mode – projections onto $l=2$ and $l=3$ directions were shown to be a highly effective way of identifying relative equilibria [70] and periodic orbits [71] associated with rare, high dissipation ‘bursting’ events, which had eluded previous search protocols. The apparent connection between the learned embeddings and simple invariant solutions can also be exploited to accurately measure ‘similarity’ between vorticity field, something which we now examine.

**Fig. 3:**
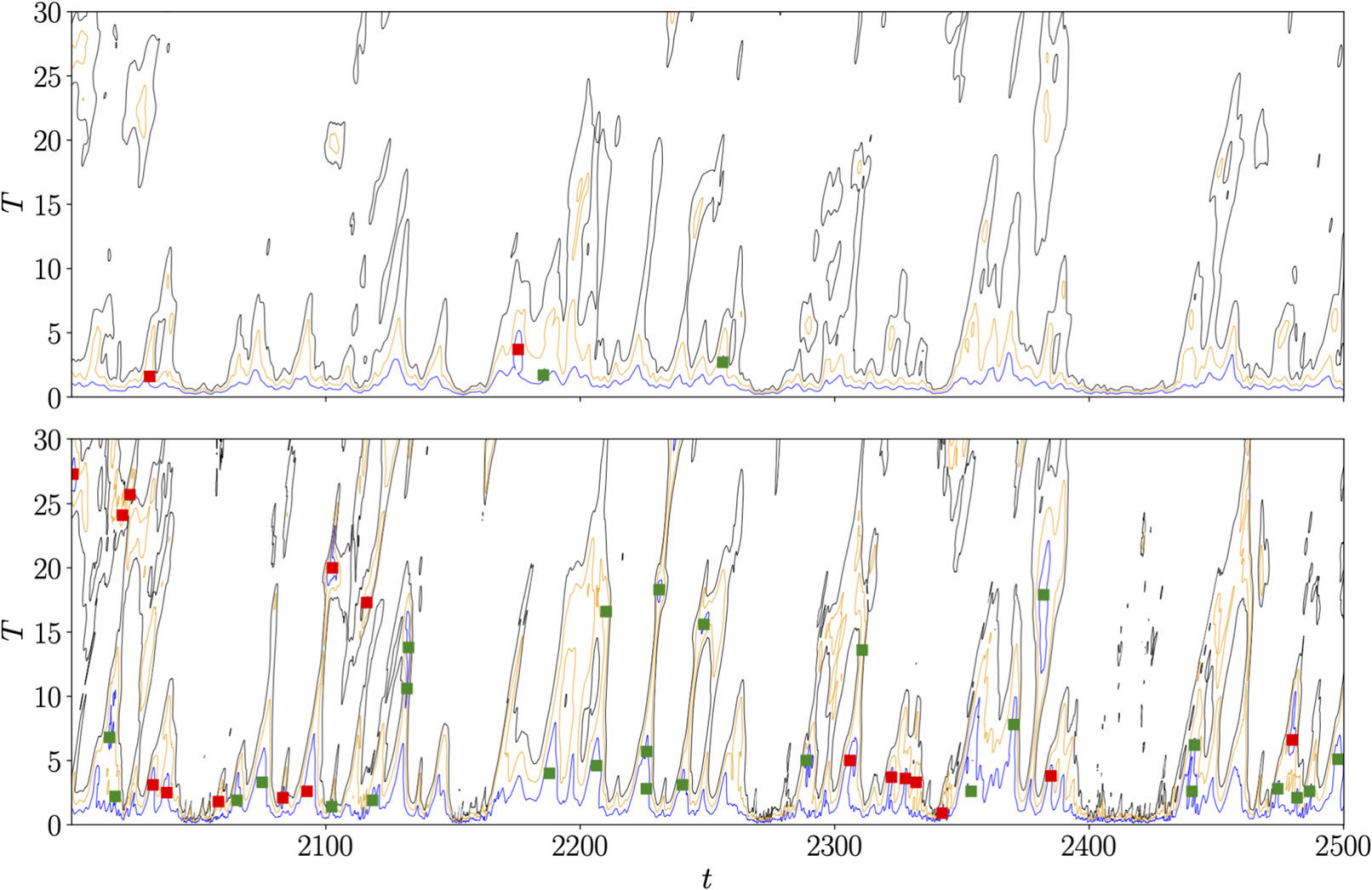
Comparison between recurrent flow analysis in physical and latent space, as originally reported in [71]. (Top) Contours of the recurrence measure (11), with blue lines indicating the threshold $R_{\omega } = 0.3$ (other lines are $R_{\omega } = 0.45$ in gold and $R_{\omega } = 0.6$ in black). (Bottom) Contours of latent recurrence measure (12), with $R_{{\mathcal {E}}} \in \{0.015, 0.03, 0.045 \}$ (lowest value is the threshold, colors again run blue, gold, black from low to high $R_{{\mathcal {E}}}$ values). In both cases, the markers identify periodic orbit guesses, with green indicating successful convergence, red failure of the Newton algorithm

### Recurrent flow analysis revisited

As described in §1, one method to construct guesses for periodic orbits is to search for near recurrence in observations from a turbulent time series. This involves searching for local minima of

11 $$\begin{aligned} R_{\omega }(t,T) = \min _{s}\frac{\Vert {\mathscr {T}}^s \omega (t+T) - \omega (t)\Vert }{\Vert \omega (t)\Vert }. \end{aligned}$$

Guesses for periodic orbits are then triplets $(\omega (t), T, s)$ for which local minima of (11) fall below some threshold. There are two issues with this approach: (a) It requires a near recurrence to occur and (b) Euclidean distance between vorticity fields is not necessarily a good way to measure similarity between snapshots.

Given the apparent connection between the learned embeddings in an autoencoder and exact solutions of the governing equations (see discussion above and [70]), [71] considered an alternative measure of recurrence,

12 $$\begin{aligned} R_{{\mathcal {E}}}(t,T) = \min _{\alpha }\frac{\Vert \varvec{\psi }({\mathcal {E}}(\omega (t+T)) - \varvec{\psi }(\mathcal E(\omega (t)))\Vert }{\Vert \varvec{\psi }({\mathcal {E}}(\omega (t)))\Vert },\nonumber \\ \end{aligned}$$

where $\varvec{\psi }$ is a vector observable whose elements consist of the absolute value of projections onto SVD modes within latent Fourier eigenspaces:

13 $$\begin{aligned} \varvec{\psi }(\omega ) := \begin{pmatrix} ({\textbf{u}}_{l=0}^1)^H {\mathcal {P}}^{0}({\mathcal {E}}(\omega )) \\ ({\textbf{u}}_{0}^2)^H {\mathcal {P}}^{0}({\mathcal {E}}(\omega )) \\ \vdots \\ |({\textbf{u}}_{1}^1)^H {\mathcal {P}}^{1}({\mathcal {E}}(\omega ))| \\ \vdots \\ |({\textbf{u}}_{3}^{d(3)})^H {\mathcal {P}}^{3}({\mathcal {E}}(\omega ))| \end{pmatrix}, \end{aligned}$$

with ${\mathcal {P}}^l({\mathcal {E}}) := \sum _{k=1}^{d(l)}\mathcal P^l_k({\mathcal {E}})$, and ${\textbf{u}}^j_l$ is the $j^{\text {th}}$ PCA mode of the embeddings projected onto the *l*-eigenspace. The observable, $\varvec{\psi }$ is invariant to streamwise shifts of the physical vorticity field, and estimates for the shift associated with the relative periodic orbit are obtained from the phase difference in projections onto the leading mode within the $l=1$ subspace (see [71]).

A comparison of the two recurrence measures (11 and 12) is reported in figure 3 for an example turbulent time series at $Re=40$. The ‘standard’ threshold $R_{\omega }=0.3$ [15, 60, 62] is used in physical space to search for periodic orbits (note the relatively high value required to generate guesses), yielding two successful convergences (green squares) and two failures (red squares) from the five hundred advective time units of data.

The threshold on the embedded near recurrence (12) was selected by averaging $R_{{\mathcal {E}}}$ computed from a set of physical space guesses where $R_{\omega }\approx 0.3$, for which ${\overline{R}}_{{\mathcal {E}}} \approx 0.015$. The guesses yielded from the embedded recurrence measure are much more numerous, and yield a much larger set of unstable periodic orbits – see the green squares in the lower panel of figure 3. Notably, the latent recurrence flags good guesses for periodic orbits which would have been absent even if the physical space threshold were relaxed substantially.

This approach was conducted in [71] over a much longer window of 8000 advective time units. The physical space recurrence produced a a total of 73 solutions (20 unique) with a Newton success rate (#convergences/#guesses) of $\sim 0.26$. In contrast, the embedded recurrence resulted in 543 convergences (67 unique solutions), with a success rate of $\sim 0.4$. What is particularly remarkable is that a physical-space recurrent flow analysis of more than ten times as much data performed by Chandler & Kerswell [15] produced only 50 unique solutions (same threshold on $R_{\omega }$ and the same value of $Re=40$).

While the latent-space embedding is more effective at measuring near recurrence, its use to detect near recurrence still requires the turbulent orbit to shadow a periodic solution for a full period. We now discuss a gradient-based method that removes this restriction.

## Gradient-based optimization

A critical computational component within deep learning is high-dimensional optimization for non-convex loss functions [10, 54]. This development has largely been driven by the training of large neural networks.

Neural networks trained in an ‘offline’ setting have enjoyed successful application in fluid dynamics in a number of areas, including turbulence modeling (e.g. [56]) and super-resolution of under-resolved data [25, 26] – see the review [10] for a good overview of the field. In the context of turbulence modeling, for instance learning subgrid scale stresses for use in large eddy simulations, one major disadvantage of of this approach is that deployment of a model trained offline can lead to numerical instability when incorporated into a solver.

One method that has been found to alleviate this affect is *online* training, in which the model (the neural network) is called in a solver which is used in the evaluation of the loss function. This approach, which has been dubbed ‘solver in the loop’ [59, 82] relies on the use of a differentiable flow solver to enable back-propagation of derivatives with respect to model parameters through the time-forward-map. Gradients are computed to machine precision via ‘automatic differentiation’ (AD), an approach that involves computation of the gradients via application of the chain rule through the computational graph [6].

The ‘solver in the loop’ approach was used in [50] to train a model to produce finite difference stencils for derivative evaluation which depend on the *local* velocity field. The resulting learned, ${\textbf{u}}$-dependent stencils were then used to perform a DNS-accurate calculation at $8\times$ downsampling in each spatial coordinate. The solver developed in that work, JAX-CFD [21, 50], is open source and is used in this work to search for periodic orbits. Other successes of the approach include the design of stable LES parameterizations [59] and the training of networks to perform ‘super-resolution’ without access to a high resolution reference dataset [69].

In the context of dynamical systems, a differentiable solver can be a powerful tool in the hunt for simple invariant solutions. To demonstrate the general idea of AD, consider the problem of finding an initial condition of a given energy which leads to maximum energy growth at some time *T* later. This optimization is used to search for minimal seeds in bistable systems [45] (i.e. the weakest perturbation to the laminar state that leads to breakdown to turbulence).

To determine the most dangerous disturbance of a given energy, one would seek to maximize an objective functional of the form

14 $$\begin{aligned} {\mathscr {L}}_E({\textbf{u}}_0; E_0, T)&= \frac{1}{2}\langle |\varvec{\varphi }_T({\textbf{u}}_0)|^2\rangle _V + {\lambda \left( E_0 - \frac{1}{2}\langle |{\textbf{u}}_0|^2\rangle _V\right) } \nonumber \\&{+ \int _0^T \langle {\textbf{u}}^{\dagger }\cdot [\partial _t\textbf{u}- {\textbf{f}}_{NS}({\textbf{u}}, p)] \rangle \, dt} \nonumber \\&{+\int _0^T\langle p^{\dagger } \; \varvec{\nabla }\cdot \textbf{u}\rangle \, dt } , \end{aligned}$$

where $\langle \bullet \rangle _V$ is a volume average, $\textbf{f}_{NS}$ contains the remaining terms in the Navier-Stokes equations (1) and $\lambda$, ${\textbf{u}}^{\dagger }$ and $p^{\dagger }$ are Lagrange multipliers – the latter two being commonly referred to as ‘adjoint’ fields. The constraints enforced are that (i) the energy of the initial condition is $E_0$ and (ii) that the governing equations are satisfied in space and time. Taking gradients leads to the use of adjoint equations to backpropagate derivatives to update the initial condition. The procedure requires the derivation and implementation of the adjoint system, and the derivation of ‘optimality’ conditions which relate gradients to the adjoint field at $t=0$ and depend on the specific form of the objective functional. Note that when updating initial conditions via an expression like ${\textbf{u}}_0 \leftarrow {\textbf{u}}_0 + \varepsilon \varvec{\nabla }_{{\textbf{u}}_0} {\mathscr {L}}_E$, one must still take care to project the update onto a surface of constant energy $E_0$.

In contrast, in an approach using a differentiable solver, an objective function can be written down in a much simpler form (i.e. without the constraints):

15 $$\begin{aligned} {\mathscr {L}}_E({\textbf{u}}_0; E_0, T) = \frac{1}{2}\langle |\varvec{\varphi }_T({\textbf{u}}_0)|^2\rangle _V. \end{aligned}$$

Gradients $\varvec{\nabla }_{{\textbf{u}}_0}{\mathscr {L}}_E$, which must be backpropagated through repeated iterations of the timestepper, are computed via AD to machine precision (compuation can be performed in a single line of code in the JAX library [9]) and the ${\textbf{u}}$-evolution satisfies the governing equations by design: the solver is called to evaluate the loss. The advantages of the approach are (i) its simplicity to implement, (ii) the ease with which the loss can be changed without the need for re-deriving optimality conditions and (iii) the potential to couple the approach to neural network training. We now outline the use of these ideas in the search for new simple invariant solutions.

### Vortex crystals

As a first example, consider the determination of relative equilibria in a system of *N* equal-circulation point vortices. While there is a great deal of literature on the determination of such structures analytically for small *N* (e.g. see the review [2]), asymmetric crystals with $N\gg 1$ are typically sought numerically. The example presented here is the determination of free-energy-minimizing states in a rotating disc. This configuration is relevant to the formation of quantized vortices in superfluids [13, 33, 85], where a sequence of dissipative transitions between meta-stable crystals is seen experimentally en route to the minmizing state.

The (complex) positions of the equal-circulation vortices $\{z_{\alpha }\}_{\alpha =1}^N$ in a disc at $|z| = 1$ and viewed in a rotating frame evolve according to

16 $$\begin{aligned} \dot{{{\overline{z}}}}_{\alpha }= &   -\frac{i}{2\pi }\left( \sum _{\beta =1}^N {}^{'} \frac{1}{z_{\alpha } - z_{\beta }} - \sum _{\beta =1}^N \frac{1}{z_{\alpha } - 1/{\overline{z}}_{\beta }}\right) + i \omega {\overline{z}}_{\alpha },\nonumber \\ \end{aligned}$$

where lengths have been non-dimensionalised by the dimensional disc radius, *R*, and time has been non-dimensionalised by $R^2 / \varGamma$, where $\varGamma$ is the circulation of each vortex. The first sum in (16), which neglects the singular term $\alpha =\beta$, is the induced velocity on vortex $\alpha$ from all the other point vortices in the disc, while the second sum is the velocity induced by the image vortices. The dimensionless rotation rate is denoted by $\omega$.

Predicting the free-energy minimizing state at a given value of $\omega$ requires a search over all possible *N*. Campbell & Ziff [13] showed that the problem can be simplified by justifiable neglect of the image vortices. For crystals this approximation is acceptable beyond the critical angular rotation rate at which the relative equilibrium first appears, and becomes increasingly valid as $\omega$ is increased and the vortices bunch closer to $z=0$. In the absence of images, the appropriate definition of the free energy is

17 $$\begin{aligned} {\mathscr {F}}_0= &   -\sum _{\alpha < \beta } \log |z_{\alpha } - z_{\beta }|^2 - 2 \pi \omega \sum _{\alpha } (1- |z_{\alpha }|^2).\nonumber \\ \end{aligned}$$

The angular rotation rate can now be set arbitrarily, and [13] showed that it is possible to define an $\omega$-independent label for the crystals which is the difference between (17) and a ‘continuum’ approximation to the free-energy, $\varDelta f:= {\mathscr {F}}_0(N,\omega ) - {\mathscr {F}}_C(N, \omega )$ (for full details see [13, 17]).

Campbell & Ziff [13] then found the free-energy minimizer and a handful of other low-energy states for various $N\le 50$ via gradient descent on the free-energy itself. The utility of an augmented loss function which involves unrolling vortex trajectories was then demonstrated in [17], in which a loss of the following form was considered:

18 $$\begin{aligned} {\mathscr {L}} := \kappa \; {\mathscr {F}}_0 + (1 - \kappa )\left( {\mathscr {L}}^T_{I}+{\mathscr {L}}^{T/2}_{I}\right) , \end{aligned}$$

where

19 $$\begin{aligned} {\mathscr {L}}^T_{I} := \frac{\mathop {\sum }_{\alpha< \beta } | \ell _{\alpha \beta }(T) - \ell _{\alpha \beta }(0) |^2}{\mathop {\sum }_{\alpha < \beta } | \ell _{\alpha \beta }(0) |^2}, \end{aligned}$$

with $\ell _{\alpha \beta }(t) = \Vert {\textbf{x}}_{\alpha }(t) - {\textbf{x}}_{\beta }(t)\Vert$, the distance between vortices $\alpha$ and $\beta$. This extra contribution, which is added to search explicitly for relative equilibria, massively expands the library of crystals that can be found. Minmizing ${\mathscr {F}}_0$ alone tends to yield only the lowest energy state.

The loss (18) is minimized via gradient based optimization using an Adam optimizer [49], and the relative importance of the additional, trajectory-dependent term is adjusted by incrementally decreasing the parameter $\kappa$ from $\kappa =1$ to $\kappa =0$ over the course of the optimization. The integration time is set at $T=10$, and the vortex positions are rescaled at each optimization step such that average rotation rate around the origin is $\pi / 2T$. Gradients with respect to the initial conditions are computatable in a single line of code owing to the underlying differentiability of the timestepper. The vortices are initialized randomly and optimisations yielding $\mathcal L^T_I\le 10^{-3}$ are passed to a Newton solver for convergence.

**Fig. 4:**
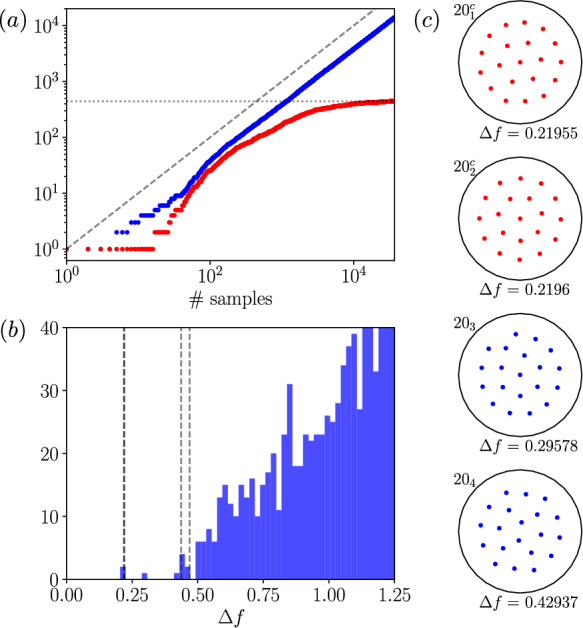
Relative equilibria in a system of $N=20$ equal-circulation point vortices, found via gradient-based optimization, adapted from results reported in [17]. (a) Number of convergences (blue) and number of unique convergences (red) measured against number of attempts. Dashed diagonal line indicates a linear scaling, while the horizontal line identifies the final number of unique solutions (441). (b) Histogram of states against the free-energy (here the rotation-rate-independent measure $\varDelta f$ is used, see text). The solutions computed by Campbell & Ziff [13] are identified with vertical dashed lines, $\varDelta f \in \{0.21955, 0.21960, 0.43685, 0.46897\}$, and the bin width for the histogram is $\varDelta f = 0.025$. (c) Vortex positions for the four lowest-energy states. Red markers indicate that the state belongs to one of a continuous family of solutions (distinct from the group orbit corresponding to axial rotation)

A summary of optimizer performance and results for $N=20$ vortices is reported in figure 4. In panel 4(a) the raw number of solutions converged is plotted against the number of random initial conditions, and vortex crystals are converged at an approximately constant rate. Tracking the number of unique solutions (red symbols) indicates diminishing returns after a large number of runs, with a suggestion that the number of unique crystals is saturating. The final number of low-energy states (441) is two orders of magnitude larger than the number of states obtained in [13] and includes many low energy states in the vicinity of the global minimum that were missed in that early work – this can be seen clearly in the histogram of states reported in panel 4(b), where previously known crystals are highlighted with dashed lines. At several other values of *N* considered in [17] new global minima were also identified.

The four lowest-energy states from the $N=20$ computation are shown in figure 4. Intriguingly, for some values of *N* (including $N=20$ shown here) many apparently ‘unique’ crystals were found with identical free energies, though on further analysis these were found to belong to a continuous family of solutions (separate from the simple continuous rotational symmetry). Two examples of crystals with this property are highlighted with red symbols for the vortices in figure 4(c). Continuous families of crystals were found to be restricted to double-ringed configurations, and movement through the continuous family corresponds (roughly) to a smooth motion of the outer ring with the inner ring held fixed. The work in [17] also went further to compute homoclinic orbits attached to unstable crystals (also via gradient-based optimization and AD) and to find non-dynamical energy-minizing pathways from nearby stable equilibria to the global ${\mathscr {F}}$-minimizer.

### Application to turbulence

The application of this approach to search for dynamically relevant periodic orbits in a turbulent flow was demonstrated in [73] with a dramatic increase in solution discovery, similar to the vortex crystal problem.

**Fig. 5:**
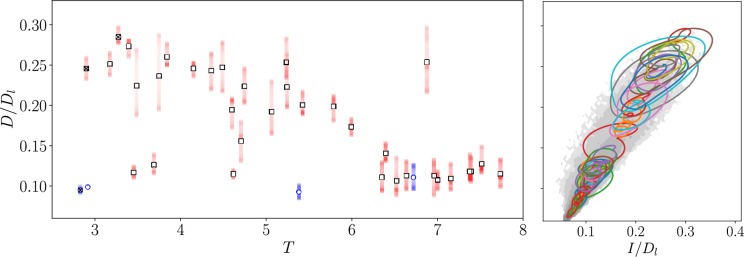
Summary of periodic orbits with periods $T<8$ found with gradient-based optimization at $Re=40$, as originally reported in [73]. (Left) Periodic orbits are visualized in terms of their dissipation rate and period, with the markers indicating the average dissipation. Solutions identified in blue were known in [15]. (Right) Visualization of the same periodic orbits in the production-dissipation plane (closed curves). Gray contours are the turbulent PDF, and are spaced logarithmically

Earlier searches for periodic orbits in the Kolmogorov flow, which have relied on some form of recurrent flow analysis (see above), have tended to produce low-dissipation orbits which consistently yield poor predictions for statistics with various weighting strategies [15, 62]. Moreover, there are relatively few examples of ‘short’ periodic solutions with the possibility that a Newton solver tends to converge to a subset of the possible solutions while missing dynamically relevant structures.

The approach of [73] was to explicitly target short-period and high-dissipation solutions via the inclusion of new terms in the loss function. Two loss functions considered were

20a $$\begin{aligned} {\mathscr {L}}_T({\textbf{u}}_0, T, \alpha )&:= \frac{\Vert \mathscr {T}^{\alpha }\varvec{\varphi }_T({\textbf{u}}) - {\textbf{u}}\Vert }{\Vert \textbf{u}\Vert } + \gamma \, (T - T^*)^2, \end{aligned}$$

20b $$\begin{aligned} {\mathscr {L}}_D({\textbf{u}}_0, T, \alpha )&:= \frac{\Vert \mathscr {T}^{\alpha }\varvec{\varphi }_T({\textbf{u}}) - {\textbf{u}}\Vert }{\Vert \textbf{u}\Vert } + \kappa \, \sigma \left( \frac{D^* - \langle D \rangle _T}{\delta }\right) . \end{aligned}$$

In both losses, the first term is simply the relative error after time marching a period, *T*, and shifting an amount $\alpha$ through the continuous symmetry (translation in *x*). Both the period and shift are determined as part of the problem. Implementation is via JAX-CFD [21, 50] for the flow solver and the ‘equinox’ library to enable the variable-*T* calculations in the loss [46].

In loss (20a) the second term targets solutions with a specific period $T^*$ – the hyperparameter $\gamma = 0.01$ is relatively weak to allow for modest departures of the period from the target without large penalization. In loss (20b) the second term is a sigmoid function which ‘activates’ if the time-average dissipation rate, $\langle D \rangle _T$, is below a specified threshold $D^*$. This loss is designed to specifically target high-dissipation cycles, with hyperparameters $\kappa = 100$ and $\delta = 0.01$.

In both cases, an AdaGrad optimizer [22] is employed. Optimization is halted when the loss drops below ${\mathscr {L}} = 0.01$, and the output is passed to a spectral Newton solver – if convergence occurs, it usually does so in a few Newton steps. One subtlety in the approach is that the optimization can introduce a finite mean vertical velocity through the domain, which changes the Kolmogorov problem (see discussion in 2). This is removed via an additional optimization seeking to slowly deform the solution to one with $V=0$, and/or is removed completely in the spectral Newton solver which sets average $V=0$ exactly.

At $Re=40$, where past work [15] had found three solutions with $T<8$, these two loss functions were minimized in [73] starting from arbitrary turbulent initial conditions. The target period in loss function (20a) was varied between $2\le T^* \le 8$ in steps of 0.5, and 50 guesses were tried per period guess. In addition, a suite of three sets of 50 initial conditions were tried with loss function (20b), with average dissipation threshold of $D^*\in \{0.12, 0.15, 0.2\}$.

The results of this sweep are reported in figure 5, which shows converged periodic orbits with periods $T<8$ visualized in terms of their dissipation rate *D* ($D_l$ is the laminar value) and energy production rate, *I*. Solutions found in Chandler & Kerswell’s recurrent flow analysis [15] are highlighted in blue – there are three with $T<8$. The loss-based approach yields 35 new solutions in this range of periods, including various high-dissipation states which previous analyses had been unable to locate.

**Fig. 6:**
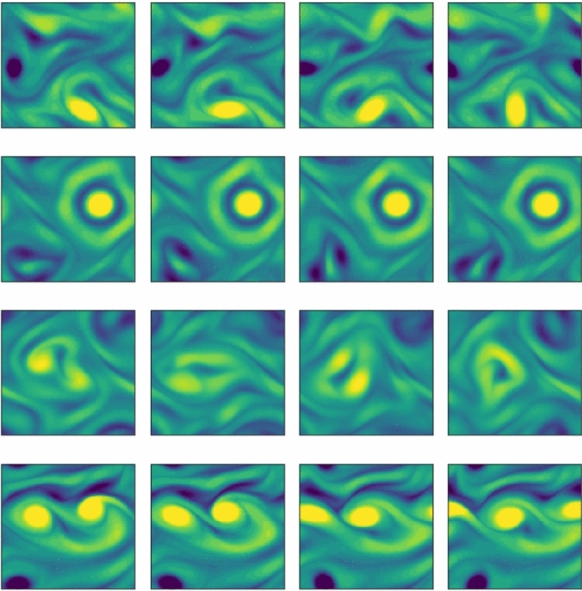
Example periodic orbits converged at $Re=100$, as reported originally in [73]. Contours are the out-of-plane vorticity are extracted at four points equispaced-in-time (left to right). From top-to-bottom properties of the solutions are: $(T, \langle D / D_l \rangle ) = (1.42, 0.06)$, (1.79, 0.05), (4.21, 0.03) and (1.16, 0.08). The contour levels run from $-10$ to $+10$ in all cases

A similar sweep was conducted at the more challenging value of $Re = 100$. Previously, $3\times 10^5$ time units of recurrent flow analysis in [15] yielded only 9 unique periodic solutions. The loss based approach – which was conducted without specific high-dissipation/period targeting – yielded 151 unique solutions, all of which appear ‘dynamically relevant’ based on their production and dissipation values (see [73]). Four example periodic orbits are included in figure 6, and exhibit a variety of vortex dynamics.

## Discussion

The utilization of neural networks in classical recurrent flow analysis, along with new gradient-based approaches, has yielded a large number of new Kolmogorov flow solutions – an order of magnitude more than have been assembled by previous methods. Here, the question of whether these solutions can be used to make statistical predictions is considered. To do this, an attempt is made to label snapshots from the turbulent attractor with the UPO which is ‘closest’ in state space, where distance is measured in the embeddings of an autoencoder.

### Statistics from structures

**Fig. 7:**
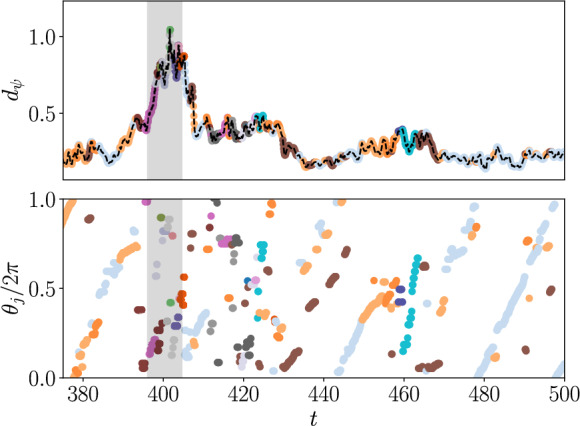
Shadowing of periodic orbits by turbulence in Kolmogorov flow at $Re=40$. (Top) Distance $d_{\psi } = \min _j d_{\psi }^j$ (see equation 22) to the nearest periodic orbit. Gray box indicates where $D/D_l > 0.15$ (a threshold for ‘high-dissipation’ events). (Bottom) Temporal around current periodic orbit, showing a monotonic increase consistent with shadowing. Markers are colored according to the nearest periodic orbit, with a random colormap selected to emphasize transitions between states

We first consider the construction of statistics at $Re=40$. An approach based on labeling according to the nearest periodic orbit was considered in [73], where distance from each periodic orbit was measured using the observable (13):

21 $$\begin{aligned} {\overline{d}}_{\psi }^j(\omega ) := \min _{m,q} \Vert \varvec{\psi }(\omega ) - \langle \varvec{\psi }({\mathscr {S}}^{(m,q)} \varphi _t(\omega _j))\rangle _{T_j}\Vert _2,\nonumber \\ \end{aligned}$$

where *j* is the index of the periodic orbit to which distance is measured and the operator ${\mathscr {S}}^{(m,q)}$ represents the application of *m* discrete shift-reflects in *y*, with or without rotation $q \in \{0,1\}$ (see discussion in [15, 73]). The current Kolmogorov configuration (four forcing waves in the box) means that an application of eight shift reflects returns the starting configuration.

In (21) the latent distance is measured relative to the *time-average* of the periodic orbit embedding, $\langle \bullet \rangle _T$. While this resulted in robust statistical reconstructions in reference [73], it does not allow one to identify/verify shadowing events in the turbulent flow. We therefore consider here the more general distance which also involves a search over the phase (in time), $\theta \in [0, 2\pi )$, around the periodic orbit:

22 $$\begin{aligned} d_{\psi }^j(\omega ) := \min _{(m,q), \theta } \Vert \varvec{\psi }(\omega ) - \varvec{\psi }({\mathscr {S}}^{(m,q)} \varphi _{\theta T_j / 2\pi }(\omega _j))\Vert _2.\nonumber \\ \end{aligned}$$

The shadowing observable (22) is plotted for a short ($\sim 125$ advective time units) turbulent trajectory in figure 7, where the line is colored according to the periodic orbit *j* which is determined to be closest. The phase around the closest periodic orbit is also plotted in the panel beneath $d_{\psi }$. While the data is quite noisy, the phase is generally observed to increase monotonically in time, consistent with a shadowing event. In most cases in figure 7 the turbulence is determined to ‘leave’ the vicinity of a periodic orbit prior to the phase completing a full $2\pi$ loop – this is consistent with the struggles of recurrent flow analysis to find many dynamically relevant solutions.

One notable feature of figure 7 is that the distance $d_{\psi }$ does not become small during a shadowing event – this is particularly notable in the ‘high-dissipation’ region (gray box in figure 7) and perhaps suggests that the actual solutions being shadowed are not in the library of converged states. Similar observations have been made when measuring shadowing of periodic orbits in Taylor-Couette flow [52].

**Fig. 8:**
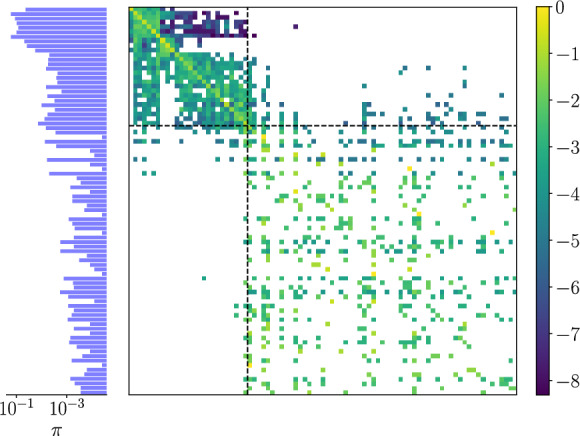
Invariant measure (left) and transition matrix (right) for periodic orbits at $Re=40$, constructed as described in the text. States in the transition matrix (rows) are ordered from lowest (top) to highest (bottom) dissipation, and the dashed lines identify the high-dissipation threshold $D/D_l =0.15$. The colors in the representation of the transition matrix are log-probabilities, with white space indicating a value of zero (a transition was never observed in $25\times 10^4$ advective time units. States which were never visited were removed from the transition matrix for clarity

The combination of periodic orbits from the loss-based search and those found using recurrent flow analysis/autoencoder projections totals to 171 unique solutions at $Re=40$. This set of solutions can be used to make robust statistical predictions when combined with the shadowing measurements. The approach can be thought of as a ‘data-driven’ alternative to periodic orbit theory [3, 4] which is appropriate for an incomplete solution library (at present there is no way to assess ‘completeness’, nor has a symbolic dynamics been found for a turbulent flow). In this spirit we seek to write an arbitrary statistic, $\varGamma$, for the turbulence as a weighted sum of the same statistic evaluated for each periodic orbit:

23 $$\begin{aligned} \varGamma ({\textbf{w}}) = \sum _{j=1}^{N_{PO}} w_j \varGamma _j, \end{aligned}$$

where the weights, which have the property $\sum _j w_j = 1$, are *the same for all statistics*.

**Fig. 9:**
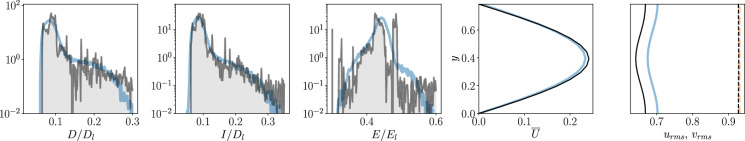
Statistics estimated from periodic orbits (black/gray) compared to ground truth data from direct numerical simulation (color) in Kolmogorov flow at $Re=40$. Weights used for all periodic orbit statistics are fixed and are set according to the invariant measure of the Markov transition matrix described in the text, $w_j \equiv \pi _j$. From left to right: comparisons of dissipation rate, production rate and kinetic energy PDFs, followed by (symmetry averaged) mean velocity profiles (black is ground truth, blue the UPO reconstruction) and root-mean-square fluctuations (solid black: ground truth $u_{rms}$, blue is the UPO reconstruction; dashed black: ground truth $v_{rms}$, orange is the UPO reconstruction)

In periodic orbit theory, the weights are determined by the Floquet multipliers of the periodic orbits. In the data-driven approach introduced in [73], which is adapted here using the new shadowing measure (22), the weights are determined from the invariant measure of a Markov chain. The set of possible states is the library of converged periodic orbits.

To determine the invariant measure, we first compute a transition matrix, ${\textbf{P}}$. To do this, a long turbulent time series (length $25 \times 10^{4}$) is stored, and the periodic orbit index *j* which minimizes (22) is computed every advective time unit, $\varDelta t =1$. The transition matrix entries, $p_{ij}$ (the probability of moving to state *j* given currently in state *i*), are then determined by simply counting the transitions $i \rightarrow j$, before normalizing such that $\sum _j p_{ij} = 1$. The invariant measure of the chain is the left of eigenvalue of $\textbf{P}$ with unit eigenvalue, $\varvec{\pi }^T {\textbf{P}} = \varvec{\pi }^T$, from which the weights are set as $w_i \equiv \pi _i$.

The transition matrix obtained in this way using the 171 unique periodic orbits at $Re=40$ is reported in figure 8, where the periodic orbits are arranged in order of increasing average dissipation rate. The invariant measure is also shown: note that many of the higher dissipation states are not visited at all – there are no transitions in or out – and these rows are absent from figure 8. Consistent with the shadowing measure in figure 7, the large probabilities on the diagonal entries of ${\textbf{P}}$ for the low dissipation states indicate that the most likely even at a given time is to remain in the vicinity of the current solution. This feature is less apparent in the high dissipation states, and trajectories appear to rapidly jump around this set of solutions. There appear to be a small number of gateway states (around the horizontal lines indicating the high-dissipation ‘threshold’ $D/D_l=0.15$) responsible for routes into/out off the high dissipation events. This behavior was also observed in the smaller library of solutions considered in [73].

A comparison is made for several statistics estimated using the periodic orbits (23) against ‘ground truth’ data from direct numerical simulation in figure 9. There is reasonable qualitative agreement in the probability density functions (PDFs), particularly when compared to earlier attempts to reconstruct statistics (e.g. see [15]). There is also coverage of high-dissipation events which were absent in earlier periodic orbit libraries. However, notable gaps remain in the PDFs – e.g. see low dissipation and higher energy values in particular. While the production, *I*, is more faithfully reconstructed here, other labeling methods/solution libraries have favored different statistics [18, 73, 77]. The first two moments of the velocity better represented, with errors on the order of a few percent are observed in the RMS profiles (contrast to early attempts to estimate statistics in [15]).

### Impact of increasing *Re*

**Fig. 10:**
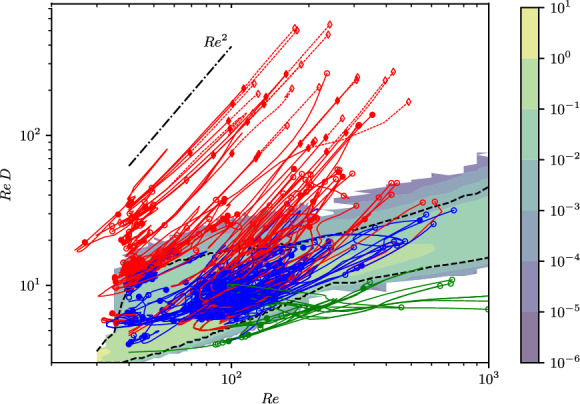
Arclength continuation of periodic orbits in *Re*, starting from solution libraries at $Re=40$ and $Re=100$, as reported originally in [18]. The dissipation rate of the solutions shown on the abscissa, with the background filled contours indicating a the PDF of the turbulence, with dashed black lines indicating the $1^{st}$ and $99^{th}$ percentiles. Curves are colored red/blue/green based on whether their ‘terminal’ dissipation rate is above/within/below this dissipation range respectively, while the continuation was stopped on the convergence of 50 states – for full details see [18]

While the increased number of states and more detailed shadowing measurements at $Re=40$ leads to a marginal increase in the already robust statistics obtained in [73], issues remain at higher *Re* despite the large number of periodic orbits found. For example, at $Re=100$, statistical reconstruction is poor despite having $\sim 17\times$ as many unique periodic orbits compared to the Chandler & Kerswell benchmark [15] – see figure 7 in [73].

One option is to expand the library of available solutions at the higher *Re* value by continuation of the library of solutions found at the lower $Re=40$. The idea that a set of solutions found at one *Re*-value can be used to make quantitative predictions at a second *Re*-value is often cited as a motivation for the effort required to identify and converge the solutions [15, 18, 20, 71].

A large Kolmogorov-continuation exercise was reported recently in [18], and resulted in an additional 101 periodic orbits at $Re=100$ to produce a library of 252 unique solutions. Despite this expansion in the number of solutions there were still notable gaps in the attempts to reconstruct turbulent PDFs, with further rapid degradation in the quality of the statistical reconstructions as *Re* was increased further.

The continuation effort in [18] is summarized in figure 10, where the time-averaged dissipation of all solutions is compared to the background turbulent PDF as *Re* is varied. The solution curves in this figure have been colored by whether they are deemed to be ‘within’ the turbulent attractor at the final *Re* value on the continuation curve – here determined simply by whether their average dissipation is between the $1^{st}$ and $99^{th}$ percentiles of the turbulent PDF. What is striking in this figure is that many solutions appear to move rapidly away from the turbulent attractor as *Re* increases (red curves are above it in terms of dissipation, green below). This raises the intriguing question as to whether these solutions were embedded within the chaotic set when they were first converged – something that has been taken for granted given their apparent similarity to the turbulence in low dimensional projections. In fact, the scaling of the various flow variables with *Re* identified in [18] is consistent with the ‘red’ solutions connecting directly to inviscid, unforced Euler solution as $Re\rightarrow \infty$, a connection also conjectured in earlier work [47, 48, 87]. The solutions being ‘nearby’ the turbulent attractor would be one explanation for the apparent distance between turbulent orbits and the periodic orbits when measuring shadowing, something which has been observed both here and elsewhere [52]. Whether these departing ‘Euler’ solutions form a chaotic saddle at finite *Re* has yet to be explored.

All of this paints a rather confusing picture for the prospects of an ECS-based reconstruction of the turbulent statistics at higher-*Re*. Solution computation is expensive: a discussion in [73] estimates $\sim 24$ hours of GPU compute time per solution at $Re=100$ starting from random initial guesses. Moreover, significant further work is then required to assess whether the structure found remains relevant at higher *Re* (or is indeed on the attractor where it was found). So, while the application of AD has generated large numbers of solutions, it may be that the ones we are still missing (perhaps longer period) lead to better statistics with fewer states – for instance, Kawahara & Kida [42] observed good agreement between turbulent/ECS statistics with a single periodic orbit. If this is the case, further ideas may be needed to generate robust longer guesses, whose convergence may be assisted by the methods described here. Assuming such solutions can be found, one would anticipate that a data-driven method like the one reviewed here will be necessary to identify appropriate weights [75, 77] due to the low chance we have of assembling a complete library of periodic orbits, while broader questions about possible non-hyperbolicity remain [38].

## Conclusions

This paper has reviewed recent machine learning-based methods for finding periodic orbits in a turbulent flow, including both how neural network architectures can enhance traditional search strategies based on near recurrence, and describing a gradient-based ‘targeted search’ approach which is accelerated with automatic differentiation and a modern optimizer. Both approaches were shown to yield large numbers of new solutions and appear to be at least an order of magnitude more effective than classic search techniques, resulting in successful convergence of hundreds of solutions where previous methods have been ineffective.

In addition, the utility of neural networks to measure similarity between flow snapshots was discussed. One consequence is an ability to determine shadowing events in turbulence, with the resulting labeling of a long turbulent trajectory leading to construction of a Markov transition matrix. The invariant measure of the associated Markov chain can be used to determine weights – in the spirit of periodic orbit theory – in an attempt to reconstruct statistics of the turbulence using the library of periodic orbits. Recent work has done exactly that, and the approach was found to be remarkably effective at modest *Re*.

### Outlook

While the new methods do appear to be robust and lead to a near-complete description of two-dimensional Kolmogorov flow at the weaker (but still chaotic) value of $Re=40$, the same is not true at higher *Re*. This is the case despite the fact that the new convergence strategies do lead to large numbers of solutions at high *Re* – the gradient-based approach in particular has resulted in hundreds of seemingly dynamically relevant new solutions.

The poor statistical performance at higher *Re* indicates that either (i) convergence of many more solutions is required, with the number of relevant solutions presumably increasing exponentially with *Re*, or (ii) the methods have not converged the dynamically relevant solutions. There are certainly hints of the latter point in figure 10 with the departure of many apparently relevant states moving rapidly away from the turbulent attractor.

One restriction of the gradient-based approach is the time-horizon of the optimization, which is limited by the Lyapunov time. This naturally restricts the effectiveness of the approach to shorter orbits, while there is some consensus that a successful use of periodic orbits to predict statistics at higher-*Re* will require longer periods [15, 42]. An approach that may have some promise here are the ‘loop convergence’ methods which do not involve time-stepping [5, 7, 53, 74]. This approach involves beginning with a closed loop in state space which does not satisfy the governing equations. One then attempts to deform the loop until it is tangent to the vector field (here $f(\omega )$ if $\partial _t \omega = f(\omega )$) at all points on the loop. The success of the method to find dynamically relevant states then relies on the generation of good initial guesses for entire loops, rather than initial conditions.

Another point worthy of consideration is that the performance and utility of the methods discussed here depends on the selection of a range of hyperparameters. This includes parameters used in neural network training (learning rates, batch size etc – see discussion in Appendix A) and in the optimization-based approaches used to identify solutions. In the latter case, there are parameters associated with specific loss functions (e.g. see equations 20a and 20b) but also the parameters associated with the optimizer (step size, momentum and other hyperparameters depending on the specific optimizer at hand). In the neural network training, the generally accepted practice in the machine learning community is to set hyperparameters empirically by running for a range of combinations (see e.g. [8]) – results do tend to depend strongly on learning rates, particularly for the autoencoders discussed here. For the optimization, in the examples reviewed here the parameters were selected based on rough heuristics and tests on small numbers of examples. It is plausible that a more considered selection would lead to increases in performance. While this is inherent empirical, one of the utilities of the approach is the ability to design and deploy custom loss functions targeting specific behavior with minimal implementation. Notably at higher *Re* a rich variety of solutions were obtained *without* additional terms in the loss.

A more significant consideration beyond hyperparameter selection is the design of the neural networks themselves. For instance, the purely convolutional architectures considered here (see references [71, 73]) significantly outperform earlier models developed for the same task but which include fully connected layers breaking translational equivariance [70]. However, one could remove the need for translational equivariance by performing a full symmetry reduction of the input data (see [57]) and in this instance fully connected models may have an advantage since nonlocal correlations may be more easily detected. Recent efforts to construct autoencoders have sought to remove some architectural hurdles in the detection of latent space dimensionality by adapting a training protocol which encourages low-rank embeddings (see discussion in §3 and [86]). Despite these advances, constructing increasingly accurate low order representations of turbulence - to exactly represent the inertial manifold - has proved challenging when compared to other nonlinear PDEs (e.g. Kumamoto Sivashinsky) and some new ideas are required. However, we have observed that the embedding structure discussed in §3, in which the network appears to represent snapshots based primarily on a known equilbrium solution, is remarkably robust to changes in architecture. For example, the results shown in figure 2 are near identical to those reported in [70] for a very different network structure.

As a final point, it is worth noting that a major caveat with conclusions drawn from the present efforts to find periodic solutions is that they have been restricted to two-dimensional turbulence. The behavior of a forced two-dimensional flow as $Re\rightarrow \infty$ is markedly different from three-dimensional shear flows, with the former dominated by a domain-filling vortex pair while the latter is increasingly multiscale [39]. We are currently deploying a variety of methods similar to those described in this article in three-dimensional flows, and hope to report on their effectiveness in the near future.

## Data Availability

No datasets were generated or analysed during the current study.

## A Neural network architectures

Details of the neural networks used to generate many of the results reviewed in this paper are summarized here. The model used primarily to generate the various figures in the main body of the manuscript, introduced in [71], is a deep convolutional autoencoders with ‘DenseNet’ layers in place of standard sequential convolutions [36].

The terminology ‘dense block’ [36, 37] refers to a series of convolutional layers where the input to each layer is concatenated with its output prior to being passed to the next convolution in the sequence. In the architecture here, all dense blocks feature three convolutional layers, each of which produces 32 ‘feature maps’. Due to the repeated concatenation this means that, if the input to the layer is of dimension $(N_x, N_y, N_c)$ ($N_c$ being the number of channels, and the batch size has been omitted for clarity), then the output of the dense block will be of shape $(N_x, N_y, N_c + 3 \times 32)$.

Periodic padding is applied upstream of every convolution so that the output retains the same horizontal and vertical dimensions as the input to the layer, and GELU activations [32] are used throughout, apart from the last layer where $\tanh$ is applied (input normalized, $\max |\omega /\omega _{norm}| < 1$.

As described in §3, the autoencoder ${\mathcal {A}}$ is a composition of encoder, ${\mathcal {E}}$, and decoder, ${\mathcal {D}}$ functions. Dimensionality reduction in the encoder is performed between dense blocks by ‘max pooling’ – retaining the max value within a feature map of a given size. Max pooling on $2\times 2$ blocks reduces the dimension by a factor of four. In the decoder, ‘upsampling’ layers – which perform a nearest-neighbor interpolation – are used between convolutions or dense blocks to incrementally return the embeddings to the original (input) shape.

The structure of the encoder can be summarized as follows:

24 $$\begin{aligned} \omega \rightarrow&\text {Conv}(8\times 8, 64) \rightarrow \text {Dense}(8 \times 8) \rightarrow \text {MP}(2,2) \rightarrow \nonumber \\&\text {Conv}(4\times 4, 32) \rightarrow \text {Dense}(4 \times 4) \rightarrow \text {MP}(2,2) \rightarrow \nonumber \\&\text {Conv}(4\times 4, 32) \rightarrow \text {Dense}(4 \times 4) \rightarrow \text {MP}(2,2) \rightarrow \nonumber \\&\text {Conv}(2\times 2, 32) \rightarrow \text {Dense}(2 \times 2) \rightarrow \text {MP}(2,2) \rightarrow \nonumber \\&\text {Conv}(2\times 2, 32) \rightarrow \text {Dense}(2 \times 2) \rightarrow \text {MP}(2,1) \rightarrow \nonumber \\&\text {Conv}(2\times 2, 32) \rightarrow \text {Dense}(2\times 2) \rightarrow \text {Conv}(2\times 2, M )\nonumber \\&\equiv {\mathscr {E}}(\omega ). \end{aligned}$$

Here, ‘Conv’ indicates a single convolutional layer with periodic padding, where the arguments are the shape of the convolutional filter $\text {width} \times \text {height}$ and the number of feature maps. The term ‘Dense’ identifies a dense block of convolutions, with each convolution adding 32 feature maps. The abbreviation ‘MP’ indicates max pooling, with the arguments being the filter dimensions. The decoder network, ${\mathcal {D}}$, is essentially the same structure in reverse, with upsampling replacing pooling operations. The network was designed for input images of shape $N_x\times N_y = 128\times 128$. Other similar architectures have been used in a similar context [69, 70] – see references for full details.

All models reviewed in this paper were trained to minimize a loss identical, or similar to, equation 4 over a large dataset of reference vorticity snapshots (typically $O(10^5)$ images). Gradient updates to the network weights are applied using the Adam optimization algorithm [49]. Network performance can be sensitive to the choice of both learning rate, $\eta$, and batch size (number of samples used to estimate an individual gradient). ‘Optimal’ values for these parameters are determined through experimentation as is standard in the machine learning literature [8]. Typically $\eta = O(10^{-4})$ for our problems, with a batch size typically set to one of $b\in \{16,32,64\}$.
